# Anatomical and Ontogenetic Basis of Silver Thiosulfate-Induced Masculinization in Genetically Female *Cannabis sativa*

**DOI:** 10.3390/plants15142153

**Published:** 2026-07-13

**Authors:** Marina D. Judkevich, María Antonia Marassi, Ana Maria Gonzalez

**Affiliations:** Instituto de Botánica del Nordeste (UNNE-CONICET), Facultad de Ciencias Agrarias, Universidad Nacional del Nordeste, Corrientes PC 3400, Argentina; marina-judkevich@hotmail.com (M.D.J.); mamarassi@gmail.com (M.A.M.)

**Keywords:** *Cannabis sativa*, silver thiosulfate, STS (silver thiosulfate), sex reversal, masculinization, floral meristem identity, staminate flower development, anther development, microsporogenesis, pollen viability, feminized seed production, correlative microscopy

## Abstract

The induction of staminate flowers in genetically female *Cannabis sativa* is widely used for feminized seed production, yet its anatomical and ontogenetic basis remains poorly understood. This study examined silver thiosulfate (STS)-induced masculinization in genetically female plants of *C. sativa* cv. ‘Pasionaria S’ using stereomicroscopy, light microscopy, confocal laser scanning microscopy, pollen viability assays, and open-pollination tests. STS redirected floral sexual identity within the conserved architecture of the female reproductive shoot. Staminate floral meristems were first detected at 7 days post-treatment, while previously initiated pistillate flowers persisted; by 21 days, the reproductive apex was dominated by staminate structures. Induced male flowers corresponded to type II unisexual flowers, with early staminal primordia and no evidence of gynoecial primordia. Three trajectories were identified: fertile male flowers with normal anther development and viable pollen, sterile flowers with collapsed anthers and disrupted microsporogenesis, and occasional mixed flowers with localized androecial expression. Pollen viability reached 54%, and open pollination confirmed seed formation. These results show that STS-induced masculinization is a staged and heterogeneous redirection of floral meristem identity.

## 1. Introduction

*Cannabis sativa* L. (Cannabaceae) is an annual dioecious species characterized by the presence of male and female flowers on separate plants and a well-defined XX/XY chromosomal sex determination system [1,2,3,4,5]. Historically cultivated for more than 6000 years across western, central, and eastern Asia, *Cannabis* has been used as a source of fiber, food, and medicinal products [6,7]. Today, it is a multipurpose crop with applications in diverse sectors, including pharmaceuticals, food, cosmetics, biomaterials, and phytoremediation [8]. This wide range of uses has driven renewed scientific and commercial interest in the species, particularly in relation to its reproductive biology.

Sex expression in *Cannabis* is regulated by genetic, chromosomal, epigenetic, and hormonal factors [9,10,11,12]. This intrinsic plasticity allows for the occurrence of monoecious and hermaphroditic individuals, which are used for producing seeds with a skewed XX chromosome ratio [2,13]. In modern cultivation systems, especially those focused on cannabinoid-rich inflorescences, female plants are preferred because they accumulate substantially higher cannabinoid levels than male plants, predominantly in the inflorescences, where glandular trichomes are highly abundant [14,15,16,17].

**Figure 1:**
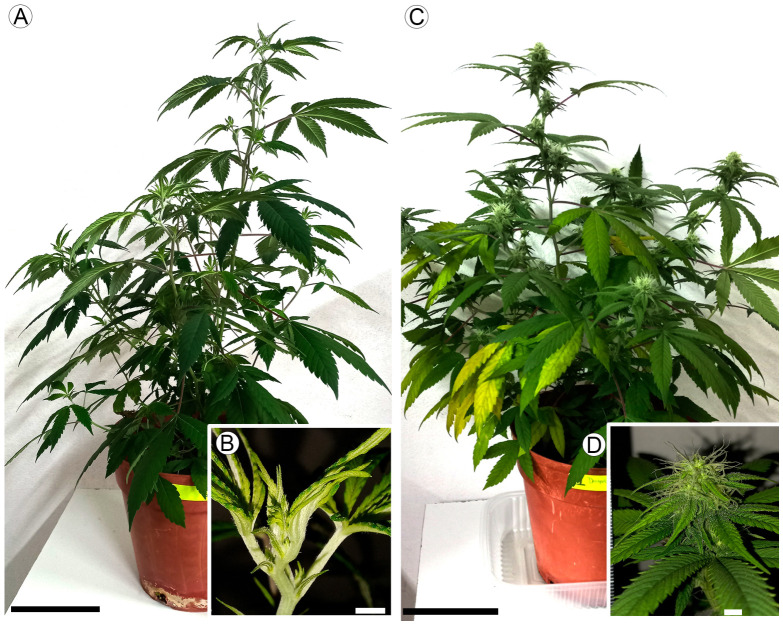
Shoot architecture of female *Cannabis sativa* under contrasting photoperiods. (**A**,**B**) Long-day photoperiods (LDPs): plant habit and detail of the shoot apex with axillary female flowers. (**C**,**D**) Short-day photoperiods (SDPs): plant habit and detail of the reproductive apex with condensed female inflorescences. Scale bars: (**A**,**C**) = 10 cm; (**B**,**D**) = 5 mm.

In both seed-propagated *C. sativa* and horticulturally propagated plants obtained from rooted cuttings, individuals grown under long-day photoperiods (LDPs) exhibit a consistent organization associated with the production of solitary axillary flowers (Figure 1A,B) [18,19,20]. The sexual expression of these early flowers is routinely used in horticultural practice to identify and retain female plants while eliminating male individuals [21]. Upon exposure to short-day photoperiods (SDPs), the shoot architecture of female plants is extensively reorganized, with increased branching, internode shortening, and leaf reduction, ultimately leading to the formation of condensed inflorescences (Figure 1C,D) [19,20,22,23].

To obtain predominantly female progenies, the *Cannabis* industry relies on the production of so-called “feminized seeds”, i.e., seeds expected to give rise mainly to female plants. These seeds are produced by crossing individuals in which both gametes originate from genetically female (XX) plants. In practice, this requires the induction of staminate flowers on selected female plants, so that their pollen can be used to fertilize other female plants. Because the resulting progeny lacks a Y chromosome, feminized seeds are expected to produce predominantly female offspring. This strategy is commonly achieved through the application of plant growth regulators or ethylene inhibitors, particularly silver-based compounds, which promote male flower development in otherwise female plants [13,24].

The chemical modulation of sex expression is not restricted to *Cannabis*. Plant growth regulators and silver-based compounds have been used to alter floral sex expression in several species, including horticultural crops such as *Cucumis sativus* (cucumber) and *Cucumis melo* (muskmelon) [25,26,27,28,29,30]. In *Cannabis*, masculinization can be achieved by increasing gibberellin activity or by inhibiting ethylene action using silver-based compounds and other anti-ethylene treatment [9,24,25,31,32]. Among these treatments, silver thiosulfate (STS) is one of the most effective methods for inducing fertile male flowers in genetically female plants and is widely applied in feminized seed production and breeding programs [13,24,30,32,33]. Although silver nitrate is highly toxic, its complexation with thiosulfate considerably reduces environmental toxicity [34,35].

Although STS-induced masculinization in *Cannabis* has already been reported, previous studies have focused mainly on treatment efficiency, number of induced staminate flowers, pollen appearance, pollen viability, pollen germination, and feminized seed production [13,24,30,33,36,37,38,39]. These studies have demonstrated the practical value of STS for sex reversal and breeding. However, the production of visible staminate flowers does not necessarily imply normal anther development, complete microsporogenesis, pollen maturation, anther dehiscence, pollen release, or reproductive functionality. Therefore, an anatomical assessment of anther development and microsporogenesis is essential for understanding the developmental consequences of STS treatment beyond visible masculinization.

Several studies have described gametophyte development, microgametogenesis, and floral ontogeny in *Cannabis* and other Cannabaceae [2,40,41,42]. However, the STS-induced transition itself remains poorly documented from an anatomical and developmental perspective.

The aim of this study was to characterize the anatomical and ontogenetic changes associated with STS-induced redirection of floral sexual identity in genetically female *Cannabis sativa* cv. ‘Pasionaria S’. Specifically, we sought to determine the timing of the sexual transition, assess whether induced staminate flowers arise through the late abortion of pistillate structures or through early specification of staminate floral identity, evaluate whether masculinization affects floral identity within the conserved female-patterned reproductive shoot architecture, identify the main developmental trajectories associated with masculinization, and examine the relationship among morphological masculinization, pollen viability, and reproductive functionality.

## 2. Materials and Methods

### 2.1. Plant Material and Growth Conditions

The study was conducted using plants of *Cannabis sativa* cv. ‘Pasionaria S’, registered in the National Seed Institute catalogue [43]. Feminized plants of this cultivar are characterized by vigorous growth, profuse branching, and long inflorescences densely covered with glandular trichomes. Its cannabinoid profile shows high cannabidiol (CBD) content (~19%) and low tetrahidrocannabinol (THC) levels (~0.5%). As a photoperiod-dependent cultivar, it requires at least 18 h of light per day to prevent floral induction.

Plants were obtained through in vitro micropropagation from nodal segments cultured on half-strength Murashige and Skoog (MS) medium without growth regulators, under controlled conditions (26 ± 2 °C, 18 h photoperiod, photosynthetic photon flux density (PPFD) of 30 µmol m^−2^ s^−1^) [44]. Ex vitro acclimatization was performed under controlled environmental conditions (26 ± 2 °C; 60–70% relative humidity) using a peat-based substrate.

After acclimatization, plants were transferred to growth chambers and maintained under long-day photoperiods (LDPs, 18 h light) using high-pressure sodium lamps (Osram Vialox, 400 W, OSRAM GmbH, Munich, Germany), providing 200 µmol m^−2^ s^−1^ PPFD. Plants were grown in Grow-Mix substrate (Terrafertil S.A., Buenos Aires, Argentina), composed of sphagnum peat, composted bark, and perlite, with 80–85% porosity, approximately 60% water retention, pH 5.0–5.8, and electrical conductivity (EC) 0.20–0.60 deciSiemens per meter (dS m^−1^), without additional fertilization. Plants were maintained under these conditions until day 60, when they reached approximately 60 cm in height and showed no evidence of compact reproductive inflorescences.

Voucher specimens (Judkevich MD 96 and Judkevich MD 97) were deposited in the CTES herbarium, Corrientes, Argentina.

### 2.2. Experimental Design

**Figure 2:**
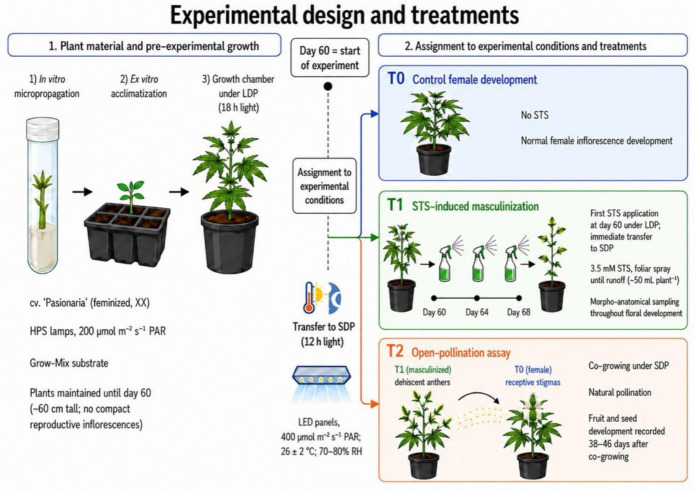
Experimental design and treatments. Schematic representation of plant material preparation, pre-experimental growth under long-day photoperiods (LDPs), change to short-day photoperiods (SDPs) at day 60, assignment to experimental conditions, control female development (T0), STS-induced masculinization treatment (T1), and open-pollination assay (T2).

Day 60 was considered the starting point of the experiment (Figure 2). At this stage, plants were assigned to the experimental conditions and transferred from long-day photoperiods (LDPs, 18 h light) to short-day photoperiods (SDPs, 12 h light) to induce reproductive development. SDP conditions were established in growth chambers equipped with LED panels (200 W; 6500 K), providing 400 µmol m^−2^ s^−1^ PPFD, at 26 ± 2 °C and 70–80% relative humidity.

Two experimental conditions were established to characterize normal female floral development and STS-induced masculinization. In addition, an open-pollination assay was performed when STS-treated plants bore dehiscent anthers, in order to evaluate the reproductive functionality of the induced pollen.

- (T0) Control female development: Untreated plants were maintained under SDPs without STS throughout the experiment, allowing for normal development of female inflorescences.
- (T1) STS-induced masculinization: Plants assigned to this treatment received the first application of STS at day 60, while still under LDP conditions, and were immediately transferred to SDP after treatment. Two additional applications were performed at 4-day intervals, on days 64 and 68, while plants were already under SDP. Silver thiosulfate was prepared from 0.1 M stock solutions of silver nitrate and sodium thiosulfate mixed in a 1:4 ratio [45] and applied at a final concentration of 3.5 mM as a foliar spray until runoff, approximately 50 mL per plant. Treated plants were sampled throughout floral development to characterize the anatomical and ontogenetic sequence of masculinization.
- (T2) Open-pollination assay: To evaluate the reproductive functionality of pollen produced by STS-induced male flowers, T1 plants bearing dehiscent anthers were maintained together with untreated T0 female plants bearing receptive stigmas. Both groups were kept under the same SDP conditions described above, allowing open pollination to occur. Fruit and seed development were recorded 38–46 days after the beginning of the open-pollination assay.

### 2.3. Morpho-Anatomical Studies

Samples from all experimental conditions (T0, T1, and T2) consisted of apical portions of shoots bearing reproductive meristems and associated floral structures at different developmental stages. During the first two weeks after STS treatment, sampling was performed every 2–4 days, three times per week, to capture key ontogenetic stages associated with the transition toward staminate floral development. At each sampling point, apical segments from seven plants per treatment were collected. Additional samples were collected at later developmental stages as needed. All samples were immediately fixed in FAA (formaldehyde: ethanol: acetic acid, 5:90:5) and used for all subsequent microscopy-based anatomical analyses (light microscopy and scanning electron microscopy).

### 2.4. Stereomicroscopy (SM)

Floral meristems and developing reproductive structures were dissected under a Leica DMLV2 stereomicroscope (Leica Microsystems, Wetzlar, Germany). Samples were manually sectioned in longitudinal or transverse orientation using a razor blade and photographed prior to histological processing when necessary. Images were acquired with a Leica Flexacam digital camera (Leica Microsystems GmbH, Wetzlar, Germany).

### 2.5. Light Microscopy (LM)

Floral apices up to 1 cm in length were processed from FAA-fixed material. Samples were dehydrated through an ascending ethanol series, pre-infiltrated with tertiary butanol, and embedded in paraffin following standard botanical procedures and a correlative multiscale microscopy framework [46,47,48]. Serial transverse and longitudinal sections (12 µm thick) were obtained using a MICROM HM325 rotary microtome (Thermo Fisher Scientific, Walldorf, Germany). Sections were stained with Safranin–Astra Blue [49], dehydrated, cleared, and mounted in synthetic Canada balsam [50].

Pollen viability was assessed using Alexander staining [51]. Fresh material was collected from ten mature flowers, including flowers with dehiscent anthers and flowers with indehiscent anthers. Pollen grains were mounted on microscope slides, stained with Alexander solution, and examined under light microscopy. Pollen grains with blue-green walls and pink-stained cytoplasm were considered viable, whereas collapsed or unstained grains were considered non-viable.

Observations and photomicrographs were obtained using a Leica DMLB2 light microscope equipped with a Leica ICC50 HD digital camera (Leica Microsystems GmbH, Wetzlar, Germany).

### 2.6. Confocal Laser Scanning Microscopy (CLSM)

Confocal imaging was performed using a Leica Stellaris 8 inverted microscope equipped with a white light laser (Leica Microsystems, Wetzlar, Germany), HC PL APO 10× dry (NA 0.4), and HC PL APO 63× oil immersion (NA 1.4) objectives (Leica Microsystems GmbH, Wetzlar, Germany). The same Safranin–Astra Blue-stained sections prepared for light microscopy were used for confocal analysis, following a correlative multiscale microscopy approach [48,52]. Fluorescence signals were detected by sequential acquisition. Excitation/emission ranges were configured for blue fluorescence at 405 nm/413–489 nm, green fluorescence at 503 nm/508–633 nm, and red fluorescence at 627 nm/635–750 nm. Optical sections were acquired in XYZ mode and assembled into z-stacks for volumetric analysis. Individual channels were merged into red–green–blue (RGB) composites using LASX Stellaris Compass software, version 5.3.0, with blue, green, and red pseudocolors assigned to the corresponding spectral detection ranges.

### 2.7. Scanning Electron Microscopy (SEM)

SEM analysis was performed using two complementary sample preparations. For surface observations, FAA-fixed floral samples, including dissected flowers, anthers, pollen sacs, and pollen grains, were used. In selected cases, internal anatomical features exposed in sectioned material were also examined by SEM. For this purpose, paraffin-embedded samples were sectioned with a rotary microtome, mounted on shortened glass slides [48], and deparaffinized in xylene.

After the corresponding preparation, all samples were dehydrated through an acetone series, critical-point dried with CO_2_ using a Denton Vacuum DCP-1 (Denton Vacuum LLC, Moorestown, NJ, USA), mounted on aluminum stubs, sputter-coated with gold using a Denton Vacuum Desk II (Denton Vacuum LLC, Moorestown, NJ, USA), and observed at 15 kV with a ZEISS EVO 15 (Carl Zeiss Microscopy GmbH, Jena, Germany) scanning electron microscope at the Electron Microscopy Service of UNNE, Corrientes, Argentina.

## 3. Results

### 3.1. Shoot Architecture, Phytomer Organization, and Female Inflorescence Development in Control Plants

**Figure 3:**
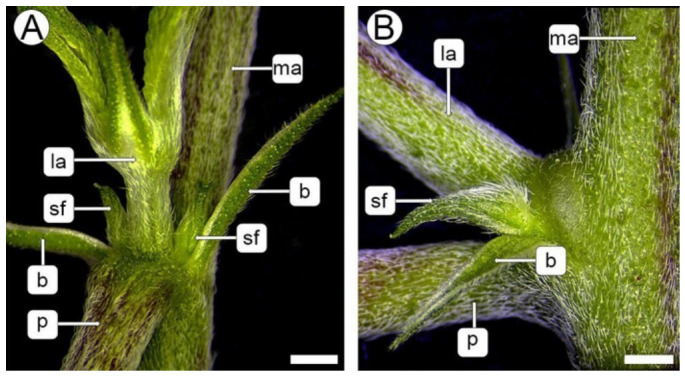
Phytomer organization (stereomicroscopy, SM). (**A**) Frontal view of a phytomer. (**B**) Lateral view of a phytomer. Abbreviations: b, bract; la, lateral axis; ma, main axis; p, petiole; sf, solitary flower. Scales: (**A**,**B**) = 1 mm.

Plants grown under long-day photoperiods (LDPs) displayed a well-defined and repetitive phytomeric organization along the main axis. Each phytomer consisted of an elongated internode bearing a large photosynthetic leaf, hereafter referred to as a fan leaf, and an axillary meristem that gave rise to a lateral shoot (Figure 1A,B). At the base of the petiole, a pair of laterally positioned bracts was consistently present, each subtending a solitary female flower in its axil, resulting in two flowers per phytomer (Figure 3A,B). Between nodes 7 and 12, the simultaneous presence of fully developed leaves, bracts, and solitary flowers indicated the transition toward the reproductive phase.

**Figure 4:**
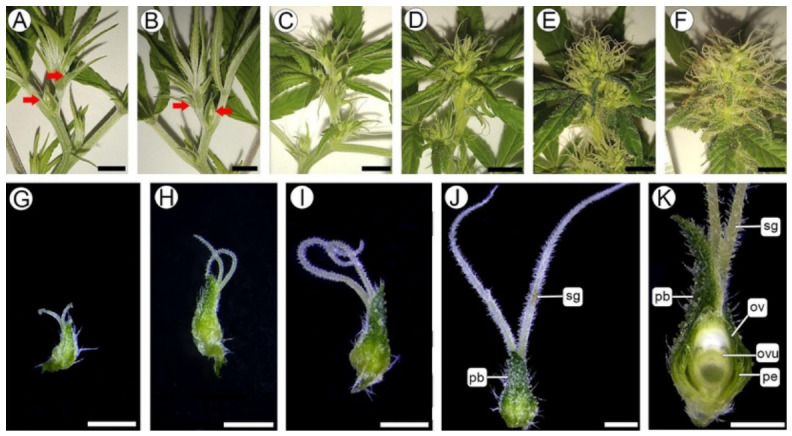
Sequence of female inflorescence development (SM). (**A**,**B**) Solitary female flowers observed 3–5 days after the transition to the short-day photoperiod (red arrows). (**C**) Young inflorescence with few female flowers at 12 days. (**D**) Inflorescence with an increased number of flowers at 14 days. (**E**) Inflorescence with most flowers at maturity at 21 days. (**F**) Mature inflorescence at 31 days, showing brown stigmas and increased trichome density. (**G**–**K**) SM. (**G**–**I**) Successive stages of female bud development. (**J**) Mature female flower with fully expanded stigma branches. (**K**) Longitudinal section of a female flower. Abbreviations: ov, ovary; ovu, ovule; pb, perigonal bract; pe, hyaline perianth; sg, stigma. Scale bars: (**A**–**F**) = 5 mm; (**G**–**K**) = 1 mm.

After the transition from long-day photoperiods (LDPs) to short-day photoperiods (SDPs), control plants (T0) showed the typical reproductive development of female *C. sativa*, characterized by a marked reorganization of shoot architecture. During the first days after the photoperiodic shift, solitary flowers were visible at the nodes, subtended by bracts and associated with axillary structures (Figure 4A,B). By 12 days, young inflorescences bearing only a few female flowers were present and remained loosely arranged along the axis (Figure 4C). At 14 days, the number of floral buds increased, and the inflorescences became more evident as internodes shortened and reproductive phytomers accumulated in close succession (Figure 4D). By 21 days, most flowers within the inflorescences had reached maturity, as indicated by the conspicuous elongation of stigmatic branches and the progressive compaction of floral units (Figure 4E). At 31 days, mature female inflorescences were fully developed, with densely packed flowers and brown stigmas (Figure 4F).

**Figure 5:**
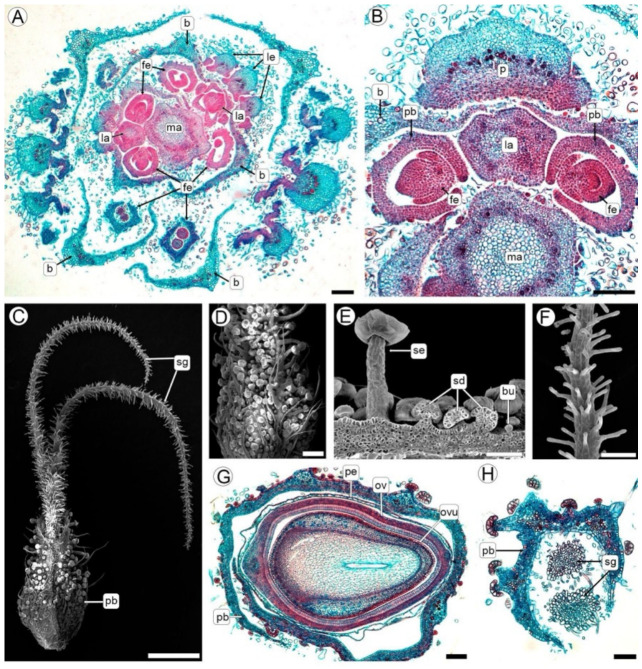
Floral apex and female flower. (**A**,**B**,**G**,**H**) Light microscopy (LM), (**C**–**F**) Scanning electron microscopy (SEM). (**A**) Cross section showing phytomer organization. (**B**) Detail of a phytomer consisting of a reduced leaf and its petiole, two opposite solitary flowers (fe) each subtended by a bract, and an axillary shoot. (**C**) Mature female flower showing the perigonal bract and elongated stigmatic branches. (**D**) Surface of the perigonal bract showing abundant glandular trichomes. (**E**) Detail of the outer epidermis of the perigonal bract with sessile capitate, stalked capitate, and bulbous glandular trichomes. (**F**) Detail of stigmatic branches. (**G**,**H**) Cross-sections through the ovary and stigmatic branches, respectively. Abbreviations: b, bract; bu, bulbous trichome; fe, female bud; la, lateral axis; le, leaf; ma, main axis; ov, ovary; ovu, ovule; p, petiole; pb, perigonal bract; pe, hyaline perianth; se, sessile capitate trichome; sd, stalked capitate trichome; sg, stigmatic branches. Scale bars: (**A**,**B**,**E**–**H**) = 100 µm, (**C**) = 1 mm, (**D**) = 300 µm.

Individual female buds showed a gradual developmental progression from small, enclosed floral buds to mature flowers with fully expanded stigmatic branches (Figure 4G–J). Each mature female flower was associated with a green perigonal bract bearing glandular trichomes (Figure 5E). Upon removal of the bract, the flower showed a hyaline perianth partially enclosing the basal region of the superior ovary. The ovary was bicarpellar and unilocular, bore two elongated stigmatic branches, and contained a single ovule (Figure 4K).

### 3.2. Anatomical Organization of Female Floral Apices in Control Plants

The internal organization of female reproductive apices in control plants under SDP was reconstructed from serial transverse sections. Despite the compact arrangement of the reproductive region, individual phytomers could be recognized by the presence of a reduced leaf with its petiole, a pair of laterally positioned female flowers, bracts, lateral axes, and associated axillary shoots (Figure 5A,B). This organization allowed the relative position of vegetative and reproductive components to be established within the condensed female inflorescence.

Female floral apices were located in the axil of a single bract and showed an early-formed, closed, unilocular ovary enclosing a single developing ovule (Figure 5B).

The mature female flower (Figure 5C–H) showed a perigonal bract composed of an outer epidermis bearing glandular (sessile capitate, stalked capitate, and bulbous) and non-glandular trichomes, as well as raised stomata; an inner epidermis with predominantly non-glandular trichomes and few bulbous trichomes; and a mesophyll formed by parenchymatic cells with dense cytoplasm, including tannin-containing cells and calcium oxalate druses. Collateral vascular bundles were present. The hyaline perianth was glabrous on both epidermal surfaces and had a mesophyll composed of a few layers of parenchymatic cells, containing five vascular bundles. The ovary wall consisted of a glabrous outer epidermis with dense cytoplasm, a palisade-like inner epidermis with conspicuous nuclei, and a mesophyll of a few layers of parenchymatic cells, including two collateral vascular bundles. The ovule occupied most of the locular cavity. The stigmatic branches had a papillose epidermis and a parenchymatic mesophyll (Figure 5C,F,H).

### 3.3. Masculinization Sequence Under STS Treatment (T1)

**Figure 6:**
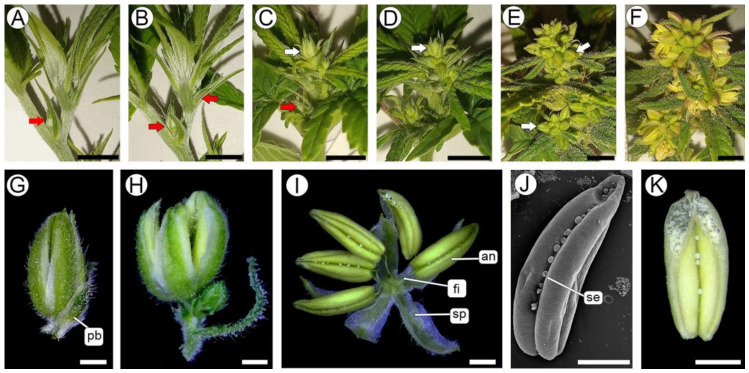
STS-induced masculinization sequence and development of male flowers (T1). (**A**–**I**,**K**) SM, (**J**) SEM (**A**) At 3 DPT and (**B**) 5 DPT, solitary female flowers were still visible at the nodes (red arrows). (**C**) At 12 DPT, coexistence of basal solitary female flowers (red arrow) and apical male buds (white arrow) could be seen. (**D**) At 14 DPT and **(E**) 21 DPT, there was a progressive increase in the number of male buds within developing inflorescences. (**F**) At 31 DPT, male inflorescences bearing buds and flowers with dehiscent anthers releasing pollen were seen. (**G**,**H**) Successive stages of male flower development. (**I**) Mature male flower showing sepals and free stamens. (**J**) Detail of an indehiscent anther. (**K**) Detail of a dehiscent anther with pollen. Abbreviations: an, anther; fi, filament; pb, perigonal bract; se, sessile capitate trichome; sp, sepal. Scale bars: (**A**–**F**) = 5 mm; (**G**–**K**) = 1 mm.

Following STS application and transfer to inductive photoperiod conditions (12 h), treated plants showed a progressive shift in floral sex expression (Figure 6). At 3–5 DPT, reproductive shoots still bore solitary female flowers comparable to those observed in control plants (Figure 6A,B). By 12 DPT, male buds became visible in apical positions, whereas previously formed solitary female flowers persisted at basal nodes (Figure 6C). From 14 to 21 DPT, the number of male buds increased progressively within the developing inflorescences (Figure 6D,E). By 31 DPT, inflorescences bore staminate flowers at different developmental stages, including flowers with dehiscent anthers releasing pollen (Figure 6F).

Induced male flowers were sessile and consisted of five sepals and five free stamens (Figure 6G–I). The stamens bore tetrasporangiate anthers with sessile trichomes in the connective region and long, slender filaments; at maturity, some anthers opened longitudinally and released pollen (Figure 6J,K). No female reproductive structures were externally evident.

During the induction process, flowers with intermediate or reduced sexual expression were also observed, including flowers with both androecial and gynoecial structures or with a reduced number of stamens. These variants are described in detail below.

### 3.4. Sexual Transition Under STS Treatment

**Figure 7:**
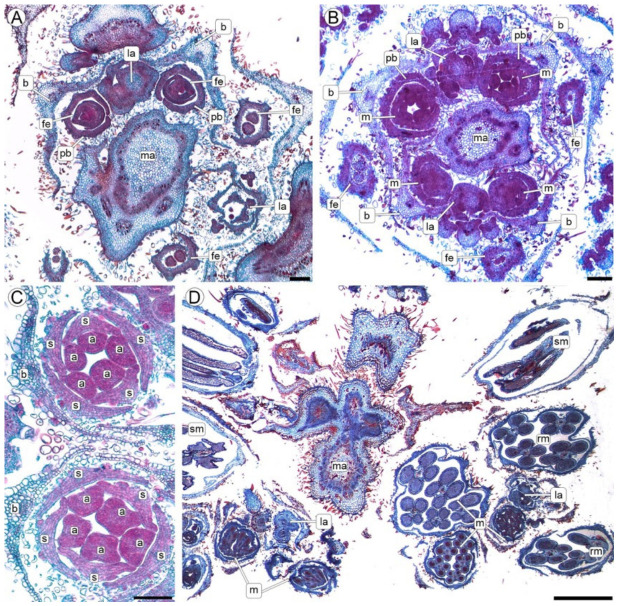
STS-induced redirection of floral sexual identity in transverse sections of the apical inflorescence at 3, 7, and 21 DPT(LM). (**A**) Reproductive apex at 3 DPT, still with female flowers. (**B**) Reproductive meristem at 7 DPT, showing the first male buds. (**C**) Detail of two developing male flowers, each subtended by one bract and showing five sepals surrounding five developing stamens. (**D**) Reproductive apex at 21 DPT, showing fertile, sterile, and reduced male buds and flowers. Abbreviations: a, anther primordia; b, bract; fe, female bud; la, lateral axis; m, male bud; ma, main axis; rm, flower with reduced masculinity; pb, perigonal bract; s, sepal; sm, sterile male bud. Scales: (**A**–**C**) = 100 µm, (**D**) = 1 mm.

At 3 days post-treatment (DPT), reproductive apices of STS-treated plants showed only pistillate floral meristems and were anatomically comparable to control apices (Figure 7A). Female flowers were associated with bracts and leaf axils and showed early gynoecium differentiation, with no evidence of staminal primordia.

By 7 DPT, the first staminate floral meristems were clearly recognizable within the reproductive apex (Figure 7B). At this stage, previously initiated female flowers were still present and continued their development, whereas the production of new female floral meristems was no longer observed. From this point onward, newly initiated flowers corresponded to male floral structures, indicating the onset of STS-induced sexual transition at the anatomical level.

STS-treated reproductive apices preserved the same basic phyllotactic and phytomeric organization described for control female inflorescences. The reproductive axis retained a compact arrangement of reiterated phytomers around the main axis, with reduced leaves, bracts, lateral axes, and floral buds occupying comparable relative positions (Figure 7B).

Staminate floral apices were characterized by the initiation of five sepal primordia followed by five staminal primordia. The latter first appeared as small dome-shaped protrusions positioned opposite the sepals and later differentiated into tetrasporangiate anthers (Figure 7C). The central region of the male floral apex was occupied by developing stamens, with no evidence of gynoecial primordia or carpel initiation.

At later stages, the reproductive apex became progressively dominated by staminate structures. By 21 DPT, only male buds were present (Figure 7D).

**Figure 8:**
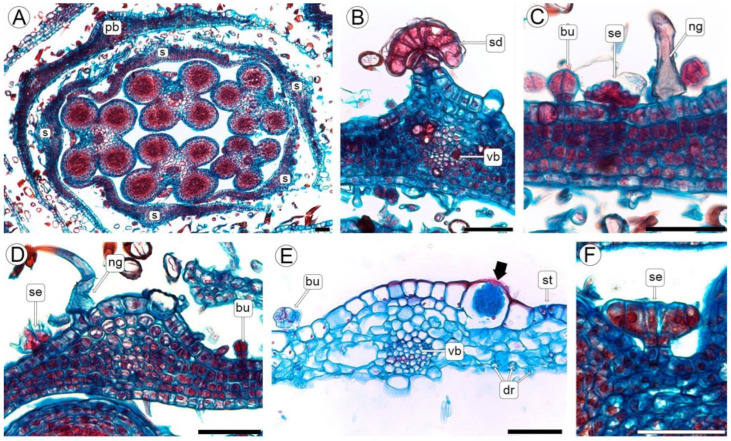
Anatomy of the male flower (LM). (**A**) Cross-sections of male flower showing the perigonal bract, sepals, and anthers. (**B**,**C**) Perigonal bract. (**D**) Sepal from a flower bud with trichomes. (**E**) Sepal from a flower at anthesis, showing druses and the base of a non-glandular cystolithic trichome (arrow). (**F**) Detail of a sessile capitate trichome of the anther. Abbreviations: bu, bulbous trichome; dr, druse; ng, non-glandular trichome; pb, perigonal bract; s, sepal; se, sessile capitate trichome; sd, stalked capitates trichome; sg, stigmatic branches; st, stomata; vb, vascular bundle. Scale bars: (**A**–**F**) = 50 µm.

Anatomically, mature male flowers were associated with a perigonal bract (Figure 8A) bearing glandular trichomes, including sessile capitate, stalked capitate, and bulbous types, as well as non-glandular trichomes (Figure 8B,C), similar to those observed on the perigonal bract of female flowers. Internally, the bract consisted of a few layers of parenchyma and collateral vascular bundles. Sepals at early developmental stages showed an anatomy similar to that of the perigonal bract (Figure 8D). At anthesis, however, glandular trichomes were senescent, stomata were fully developed, and abundant druses were observed on the inner side of the parenchyma (Figure 8E). The anthers were characterized by sessile capitate glandular trichomes on the adaxial surface of the connective tissue (Figure 8F).

### 3.5. Developmental Trajectories of the Androecium

Morpho-anatomical observations of the androecium after the establishment of male sexual expression revealed different developmental patterns. Three main floral types were identified: (i) male flowers with normal anther development and viable pollen, (ii) male flowers with collapsed anthers and disrupted microsporogenesis, and (iii) flowers with mixed sexual characteristics.

#### 3.5.1. Male Flowers with Normal Pollen Development and Viability

**Figure 9:**
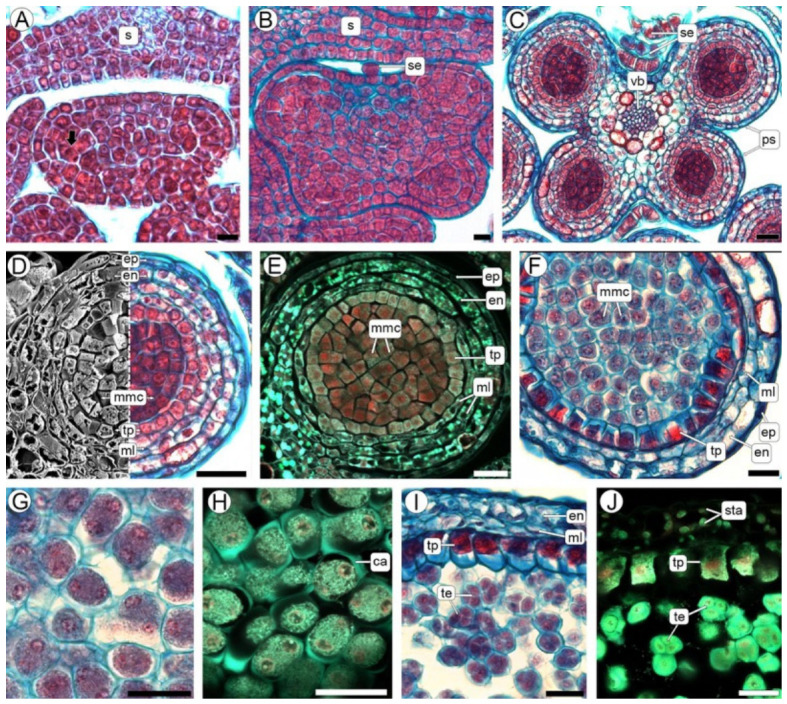
Normal development of the anther wall and microsporogenesis. (**A**–**D**,**F**,**G**,**I**) LM, (**E**,**H**,**J**) Confocal laser scanning microscopy (CLSM). (**A**) Anther primordium with mitotically active sporogenous tissue (arrow). (**B**) Anther with four defined lobes, note the young sessile capitate trichome (se) at the apex of the anther. (**C**) Tetrasporangiate anther with four distinct pollen sacs, connective tissue, and a central vascular bundle (**D**) Correlative SEM–LM detail of a pollen sac; the left half shows the SEM view and the right half shows the corresponding LM section, with all parietal layers and compact mmc. (**E**) Pollen sac with compact mmc. (**F**) Pollen sac showing the mmc at beginning of meiosis. (**G**,**H**) Details of mmc undergoing meiotic division; note the callose wall, visible by fluorescence in (**D**). (**I**,**J**) Tetrad stage, showing tetrads surrounded by callose. Abbreviations: an, anther; ca, callose; en, endothecium; ep, epidermis; fi, filament; mmc, microspore mother cells; ml, middle layers; ps, pollen sac; s, sepal; se, sessile capitate trichome; sta, starch grain; te, tetrads; tp, tapetum; vb, vascular bundle. Scales: (**A**,**B**) = 10 µm, (**C**–**J**) = 20 µm.

At the onset of anther development, young anther primordia appeared in the transverse section as ovoid masses of small, densely cytoplasmic cells undergoing active mitotic division (Figure 9A). Each primordium was associated with a corresponding sepal. After successive mitotic divisions, the anther primordium acquired a tetralobed shape (Figure 9B). In the central region, cells giving rise to the vascular bundle were distinguishable, and young sessile capitate trichomes were already visible externally in the connective region.

The young anther then differentiated into four well-defined microsporangia (Figure 9C). The wall of each microsporangium consisted of five parietal layers: the epidermis, endothecium without fibrous thickenings, two middle layers, and tapetum (Figure 9D,E). The locule contained sporogenous tissue composed of actively dividing cells.

During microsporogenesis, microspore mother cells are formed into the pollen sacs (Figure 9D,E). These cells are larger, the nucleus is more prominent and has a granular appearance, and each cell is surrounded by a layer of callose. Microspore mother cells (mmc) underwent meiotic division (Figure 9G,H), giving rise to tetrads surrounded by callose (Figure 9I,J). Subsequently, callose dissolution led to the release of individual microspores from the tetrads. At this point, the anther wall consisted of an endothecium still lacking fibrous thickenings but containing starch grains, the outermost middle layer, and a secretory tapetum. Externally, at this stage, the epidermis appeared collapsed. Subsequently, the outer middle layer also collapsed.

**Figure 10:**
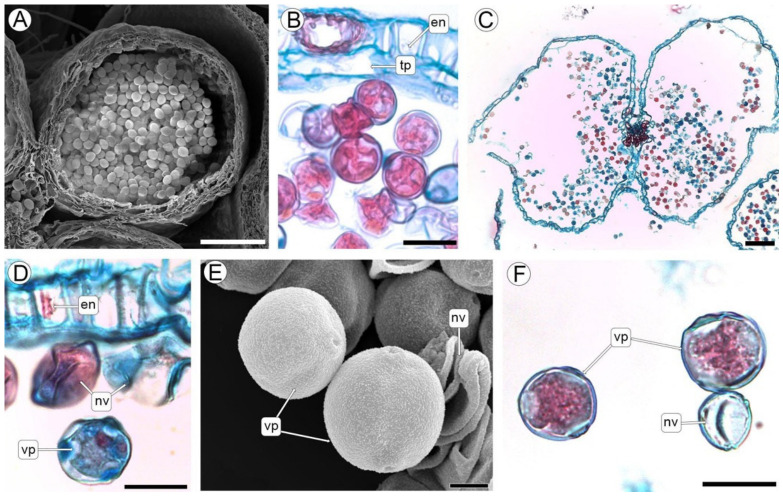
Normal development of the anther and pollen grain. (**A**,**E**) SEM, (**B**,**D**,**F**) LM. (**A**) Pollen sac containing free microspores. (**B**) Detail of the pre-dehiscent anther wall, showing the disintegrated tapetum and endothecium. (**C**) Dehiscent anther. (**D**) Detail of the endothecium with fibrous thickenings and pollen grains. (**E**) Viable and non-viable pollen grains. (**F**) Viable and non-viable pollen grains subjected to Alexander staining. Abbreviations: en, endothecium; nv, non-viable pollen; tp, tapetum; vp, viable pollen. Scales: (**A**,**C**) = 100 µm, (**B**,**D**,**F**) = 20 µm, (**E**) = 5 µm.

During microgametogenesis, each free microspore became vacuolated and underwent an unequal mitotic division, giving rise to a two-celled immature pollen grain. At this phase, the tapetum was largely collapsed, the middle layers had degenerated, and the epidermis was strongly compressed (Figure 10A,B). As pollen maturation progressed, the endothecium developed fibrous thickenings, and the mature anther wall was reduced mainly to the endothecium and a flattened epidermal remnant (Figure 10A,B). Anthers opened by longitudinal dehiscence, releasing pollen grains (Figure 10C). However, together with apparently normal pollen, a high proportion of collapsed pollen grains was also observed (Figure 10D–F). Alexander staining showed that pollen viability reached 54% in these flowers; pollen grains with blue-green stained walls and pink-stained cytoplasm were considered viable (Figure 10F).

Within the inflorescence, approximately 20–26% of mature flowers corresponded to this floral type, which was easily distinguished by fully dehiscent anthers.

#### 3.5.2. Male Flowers with Collapsed Anthers and Disrupted Microsporogenesis and Microgametogenesis

**Figure 11:**
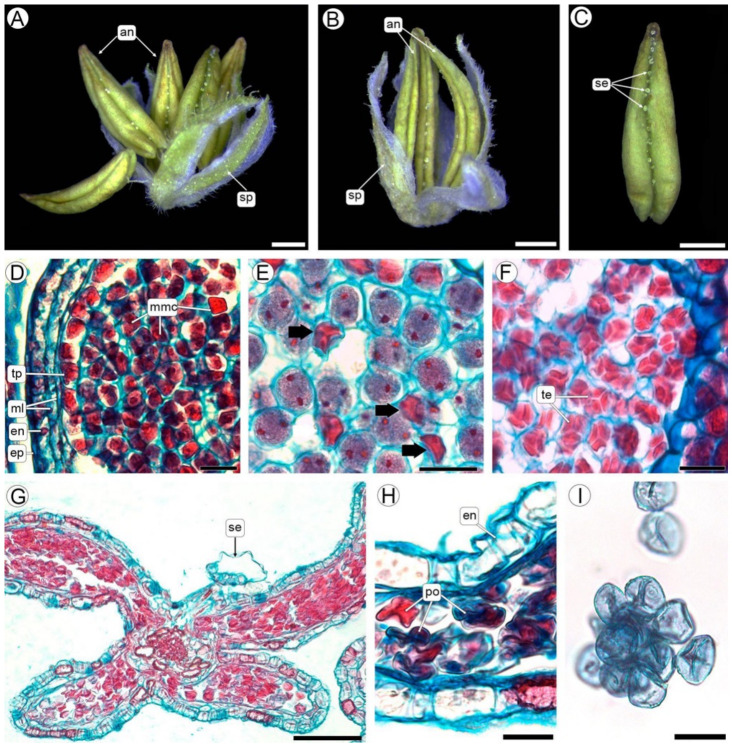
Abnormal development of the anther and pollen grain. (**A**–**C**) SM, (**D**–**I**) LM. (**A**,**B**) Flowers with reduced masculinity, showing five and three stamens with indehiscent anthers, respectively. (**C**) Detail of an indehiscent anther. (**D**,**E**) Pollen sacs with microspore mother cells showing cytoplasmic collapse (arrows), during premeiotic and meiotic stages, respectively. (**F**) Collapsed tetrads. (**G**,**H**) Indehiscent anther with mature endothecium, fibrous thickenings, and collapsed pollen grains. (**I**) Non-viable pollen grains subjected to Alexander staining. Abbreviations: an, anther, en, endothecium; ep, epidermis; ml, middle layers; mmc, microspore mother cell; po, pollen grain; se, sessile capitate trichome; sp, sepal; te, tetrads; tp, tapetum. Scales: (**A**–**C**) = 1 mm, (**D**–**F**,**H**,**I**) = 20 µm, (**G**) = 100 µm.

In this floral type, STS-induced male flowers developed stamens and anthers, but the anthers remained indehiscent (Figure 11A–C) and contained collapsed sporogenous or pollen tissue. Externally, some flowers appeared morphologically normal and bore five stamens (Figure 11A), although their anthers failed to open. Similar indehiscent anthers were also observed in flowers with reduced masculinity, characterized by a lower number of stamens (Figure 11B). Although the endothecium developed fibrous thickenings comparable to those observed in fertile anthers, dehiscence did not occur (Figure 11C,G).

Pollen development was arrested at different points during microsporogenesis and microgametogenesis, followed by cellular collapse. Arrested phases included premeiotic microspore mother cells (Figure 11D), microspore mother cells undergoing meiosis (Figure 11E), tetrads (Figure 11F), and pollen grains at different stages of development (Figure 11G,H). In the most advanced abnormal anthers, the locules contained collapsed and agglomerated pollen grains despite the presence of a mature endothecium with fibrous thickenings (Figure 11G,H). Pollen extracted from these indehiscent anthers showed a negative reaction to Alexander staining (Figure 11I).

#### 3.5.3. Aberrant Flowers with Mixed Sexual Characteristics

**Figure 12:**
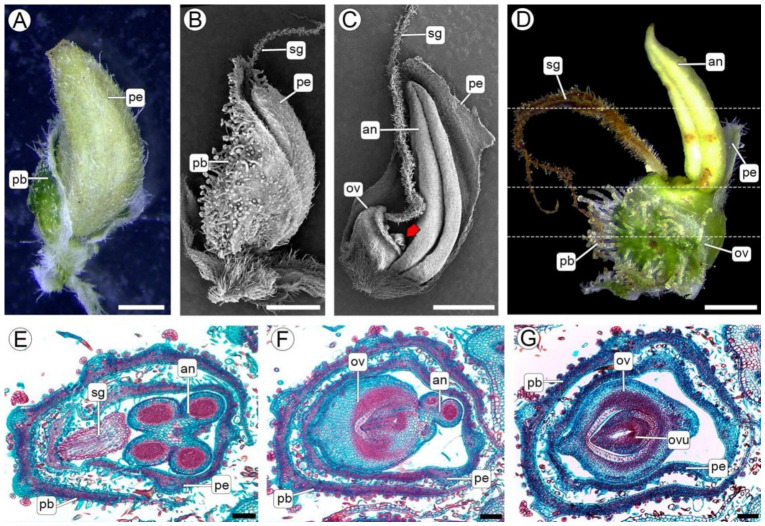
Aberrant flowers of *Cannabis*. (**A**,**D**) SM, (**B**,**C**) SEM, (**E**–**G**) LM. (**A**,**B**) Superficial view of an aberrant flower. (**C**) Same flower as in (**B**), showing the gynoecium, one developed stamen, and an atrophied stamen (red arrow). (**D**) Aberrant flower at anthesis with a gynoecium and one anther. (**E**–**G**) Transverse sections of a similar aberrant flower, at the levels indicated in (**D**), from apex (**E**) to base (**G**). Abbreviations: an = anther, ov = ovary, ovu = ovule, pb = perigonal bract, pe = hyaline perianth, sg = stigmatic branches. Scales: (**A**–**D**) = 1 mm, (**E**–**G**) = 100 µm.

Aberrant flowers with mixed sexual characteristics were occasionally recorded (Figure 12). These flowers retained a predominantly pistillate organization, including an external perigonal bract covered with trichomes, an ovary, stigmatic branches, and a single ovule. They also developed a reduced androecial structure, generally represented by one sessile anther, although additional atrophied stamens were occasionally observed.

The gynoecium consisted of an ovary bearing one (Figure 12A–C) or two stigmatic branches (Figure 12D) in an apical-lateral position. The anther was attached laterally to the apical region of the ovary by a short, inconspicuous filament. The hyaline perianth showed variable extension, ranging from partial coverage of the ovary to almost complete enclosure of the anther. Thus, these flowers combined a predominantly female floral ground plan with localized androecial expression.

Anatomically, serial cross-sections showed the relative position of the ovary, ovule, stigmatic branches, hyaline perianth, and reduced androecial structure (Figure 12E–G). Both gynoecial and androecial tissues appeared differentiated, although the anthers remained indehiscent. Because these flowers were rare and difficult to track individually, their subsequent development and capacity for fruit formation could not be assessed.

### 3.6. Functional Validation of Induced Pollen: Open-Pollination Assay (T2)

A subset of STS-treated plants (T1) developed staminate flowers with dehiscent anthers and viable pollen. When these plants were maintained together with untreated female control plants (T0) bearing receptive stigmas under inductive photoperiod conditions, open pollination was allowed to occur.

**Figure 13:**
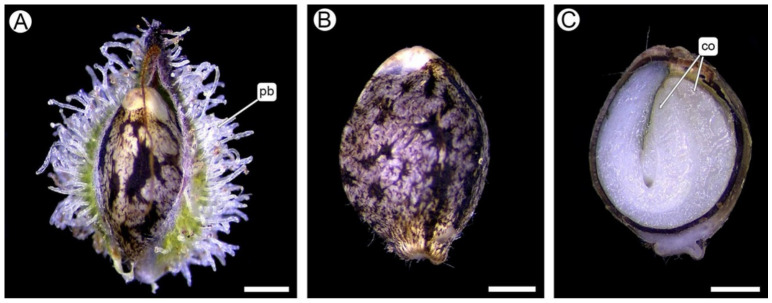
Fruit and seed development after open pollination with STS-induced male flowers (SM). (**A**) Mature fruit enclosed by the persistent perigonal bract. (**B**) Surface view of a mature seed. (**C**) Longitudinal section of a mature exalbuminous seed showing the embryo with two cotyledons. Abbreviations: co, cotyledons; pb, perigonal bract. Scale bars: (**A**–**C**) = 1 mm.

Following open pollination, fruit development was observed in the female control plants (Figure 13A). Between 38 and 46 days after the beginning of the assay, the fruits reached maturity and contained fully developed exalbuminous seeds, with embryos bearing two cotyledons (Figure 13B,C). These results confirmed that at least part of the pollen produced by STS-induced male flowers was functionally capable of fertilizing female flowers and completing seed formation.

## 4. Discussion

### 4.1. STS Redirects Floral Sexual Identity Without Altering the Female-Patterned Inflorescence Architecture

Silver thiosulfate (STS) is widely recognized as one of the most effective ethylene inhibitors for inducing sex reversal and producing feminized seeds in *C. sativa* cultivars [13,24,30,38]. The present study confirms that STS successfully induced staminate flowers in genetically female *C. sativa* cv. ‘Pasionaria S’. However, a central finding is that masculinization was restricted mainly to floral sexual identity and did not involve a broader reorganization of the reproductive shoot.

Treated plants retained the compact inflorescence architecture typical of female plants under short-day inductive conditions, including reproductive phytomers, reduced leaves, bracts, short axes, and sessile flowers. Thus, STS did not transform the female-patterned inflorescence into the lax, panicle-like architecture characteristic of genetically male plants, nor did it produce pedicellate male flowers [40]. Instead, it generated a mosaic reproductive phenotype: a female-type inflorescence bearing newly induced sessile staminate flowers.

This distinction is developmentally relevant. In *C. sativa*, sexual dimorphism involves both inflorescence architecture and floral organ identity, but these components appear to respond differently to STS. Female inflorescence architecture in *Cannabis* is strongly influenced by photoperiod-dependent developmental programs, and recent evidence indicates that gibberellin-mediated signaling participates in the regulation of female inflorescence development and architecture under short-day conditions [19,23]. Therefore, the persistence of the compact female-patterned inflorescence in STS-treated plants suggests that shoot-level architecture is established by developmental signals that are not overridden by the masculinizing treatment.

The anti-ethylene activity of STS provides a physiological basis for its effect on floral sex expression. In *Cannabis*, inhibition of ethylene perception by silver-based compounds is associated with the induction of male flowers in genetically female plants [13,30,38]. Studies in other crop systems have shown that STS can affect reproductive development through action on shoot-apex tissues, where silver accumulation and treatment localization support the shoot apex as an important target [53]. In the present study, however, STS did not alter the overall architecture of the reproductive shoot; instead, it redirected the sexual identity of floral meristems within a conserved female-patterned inflorescence.

This interpretation is supported by the temporal sequence observed after treatment. Previously initiated pistillate flowers persisted, whereas later-formed floral meristems developed as staminate flowers. Therefore, STS does not appear to convert already differentiated female flowers into male flowers. Rather, it redirects the sexual fate of newly formed or still-plastic floral meristems within a conserved female architectural framework.

Thus, STS should not be interpreted as inducing a complete male phenotype at the plant or inflorescence level. Its main developmental effect is the re-specification of floral sexual identity within the constraints of a pistillate-patterned reproductive shoot.

### 4.2. STS-Induced Staminate Flowers Correspond to Type II Unisexual Flowers

The anatomical evidence also clarifies the developmental nature of STS-induced staminate flowers. Mitchell and Diggle [54] distinguished two major developmental categories of unisexual flowers in angiosperms: *type I flowers*, in which both sexual whorls are initiated and one is later arrested or aborted, and *type II flowers*, in which only one sexual whorl is initiated from the beginning. This conceptual distinction has been reinforced in broader reviews on the development and evolution of unisexual flowers [55].

Based on our observations, the STS-induced male flowers of *C. sativa* cv. ‘Pasionaria S’ corresponds more closely to type II unisexual flowers. In fully masculinized flowers, floral meristems initiated perianth and staminal primordia, but no gynoecial primordia, ovary, style, stigmatic branches, or ovule were detected. Therefore, these flowers do not appear to result from the late abortion of an already initiated female whorl. Rather, STS redirects the early developmental fate of responsive floral meristems before gynoecial organogenesis begins.

This interpretation is consistent with developmental studies in Cannabaceae showing that floral reduction and unisexuality in *Cannabis* are established early during floral development [40]. In the present material, fully masculinized flowers followed a coherent staminate developmental program, whereas mixed flowers were rare and restricted to transitional stages. This supports the idea that complete STS-induced masculinization involves early sexual re-specification rather than progressive conversion of a mature pistillate floral structure.

### 4.3. Induced Staminate Flowers Can Follow the Normal Male Developmental Pathway

Once the male pathway was established, a subset of STS-induced flowers followed the main anatomical sequence expected for functional male flowers of *C. sativa*. These flowers formed five stamens with tetrasporangiate anthers and showed the typical progression of anther development, including differentiation of the anther wall layers, sporogenous tissue, microspore mother cells, meiotic stages, tetrads, free microspores, endothecial thickenings, longitudinal anther dehiscence, and pollen release.

This indicates that the induced flowers were not merely externally masculinized structures. They were capable of activating the internal developmental program required for androecial formation and pollen production. The observed sequence agrees broadly with classical descriptions of male flower development in *Cannabis*, particularly regarding the formation of tetrasporangiate anthers, a multilayered anther wall, tapetal activity during tetrad and pollen wall formation, and the subsequent maturation of the endothecium [41,42,56].

However, some cytological differences were observed. In the present material, tapetal cells were consistently mononucleate, whereas Reed [56] described tapetal cells with two, three, or four nuclei before tissue disorganization. Differences between our observations, Reed’s classical description, and more recent studies of *Cannabis* microgametophyte development may reflect variation among genotypes, cultivars, sexual phenotypes, or experimental conditions [41,42]. Therefore, STS-induced anther development follows a recognizable male pattern, but some details of anther wall dynamics may be condition- or cultivar-dependent.

### 4.4. Developmental Heterogeneity Reveals That Masculinization and Fertility Are Not Equivalent

The response to STS was heterogeneous across treated inflorescences. Three main floral trajectories were observed: staminate flowers with normal anther development and viable pollen, staminate flowers with collapsed anthers and disrupted microsporogenesis, and occasional aberrant flowers combining predominantly pistillate features with localized androecial expression. This heterogeneity indicates that floral masculinization, anther morphogenesis, pollen development, dehiscence, and reproductive functionality are related but not equivalent outcomes.

Some flowers developed apparently normal stamens and tetrasporangiate anthers, but their locules contained collapsed sporogenous tissue, arrested meiotic stages, collapsed tetrads, or agglomerated pollen grains. In other cases, the endothecium differentiated fibrous thickenings comparable to those of fertile anthers, but anther dehiscence did not occur. These observations suggest that different components of the male reproductive program may be uncoupled under STS treatment.

This distinction has also been reported in previous studies on chemically-induced masculinization in *Cannabis*. Ram and Sett [30] demonstrated that STS can induce fertile male flowers in genetically female plants, but later studies showed that pollen abundance, morphology, release, and germination may vary among genotypes and treatments. Lubell and Brand [13] reported that STS foliar sprays can consistently induce male flowers in female hemp plants, whereas DiMatteo et al. [36] showed that masculinized female genotypes may produce fewer large pollen grains and more irregular or misshapen pollen grains than genetically male plants. More recent studies also emphasize that STS responses depend on cultivar, concentration, timing, and application frequency [37,38].

Therefore, external male morphology alone is insufficient to demonstrate full reproductive conversion. A flower may acquire staminate identity and even form structurally recognizable anthers but still fail to complete microsporogenesis, pollen maturation, anther dehiscence, pollen release, or functional pollen production. Although alterations in tapetal timing or function could be involved in some of these developmental failures, this possibility cannot be confirmed from the present data and would require further ultrastructural and developmental analyses. STS efficiency should therefore be evaluated anatomically and functionally, not only by counting visible male flowers.

Although a quantitative correlation between glandular trichome senescence and anthesis was not assessed in this study, the presence and condition of glandular trichomes on bracts associated with reproductive structures may have functional and applied relevance. In *Cannabis*, glandular trichomes are the main sites of secretion and accumulation of secondary metabolites, including cannabinoids and terpenes, and are therefore central to the biological and economic value of the plant [17]. In this context, the occurrence of bracts bearing abundant glandular trichomes in association with STS-induced male flowers raises an interesting question: whether these bracts maintain a secretory profile comparable to that of bracts associated with female flowers, or whether their development and chemical composition are modified under masculinization. Addressing this point would require specific developmental, histochemical, and chemical analyses of trichome density, maturation, senescence, and metabolite composition.

### 4.5. Tapetal Dysfunction May Contribute to Pollen Collapse in Sterile STS-Induced Anthers

In fertile anthers, the tapetum is a highly active sporophytic tissue that provides nutrients, enzymes, callose-degrading activity, and materials required for pollen wall formation; its programmed cell death (PCD) is essential for normal pollen development [57].

In the present study, sterile anthers showed developmental arrest at different phases, including microspore mother cells, tetrads of microspores, and pollen at different stages of development. This pattern suggests that the male developmental pathway was initiated but not always completed. The abnormalities observed in sterile anthers may reflect disrupted coordination among tapetal activity, microsporogenesis, pollen wall formation, and pollen maturation.

Reactive oxygen species (ROS) play a key role in the temporal regulation of tapetal PCD and pollen development. In *Arabidopsis thaliana*, stage-specific ROS production by NADPH oxidases is critical for proper tapetal PCD and pollen [58], and altered ROS dynamics have been associated with abnormal tapetal degeneration and pollen abortion [59,60]. Ethylene signaling is also involved in anther development, and activation of the ETHYLENE-INSENSITIVE 2 (EIN2)-ETHYLENE-INSENSITIVE 3 (EIN3)/EIN3-LIKE 1 (EIL1) signaling pathway in the tapetum can disturb tapetal development and lead to pollen abortion [61].

Because STS interferes with ethylene perception through silver ions, one possible explanation is that excessive or prolonged silver action may disturb the physiological regulation of anther tissues. In particular, STS may affect the timing of tapetal degeneration and the cellular redox balance required for viable pollen formation. However, this mechanism remains hypothetical in the present study, because reactive oxygen species, antioxidant responses, and molecular markers of programmed cell death were not directly evaluated.

Thus, anther collapse in STS-treated flowers should be interpreted as a treatment-associated developmental instability rather than as a simple morphological failure. Future studies combining anatomical analysis with markers of tapetal PCD, oxidative stress, and ethylene-response pathways would be necessary to determine why some induced anthers complete pollen development whereas others collapse.

### 4.6. Aberrant Flowers Represent Localized Masculinization Within a Pistillate Developmental Context

Occasional intersexual flowers were observed during the transition phase after STS treatment. These flowers retained a predominantly pistillate floral ground plan, including the external perigonal bract, hyaline perianth, ovary, stigmatic branches, and a single ovule while developing localized androecial structures. Therefore, they should not be interpreted as stable bisexual flowers or as a necessary intermediate stage in the formation of induced staminate flowers.

Rather, these flowers indicate partial or localized masculinization within a female developmental context. Their occurrence supports the idea that STS acts within a narrow developmental window, affecting meristems or floral regions that remain responsive at the time of treatment. When the response is complete, staminate flowers are produced; when it is incomplete or spatially restricted, intersexual phenotypes may arise.

This interpretation agrees with previous reports of sexual plasticity in *Cannabis*. Ram and Sett [30] described female, intersexual, reduced male, and male flowers after treatment with silver nitrate or STS. Leme et al. [40] documented floral developmental diversity in Cannabaceae, including *Cannabis*, and Punja and Holmes [12] described spontaneous hermaphroditic inflorescences in marijuana, in which pistillate flowers are accompanied by anther formation. The occurrence of mixed or atypical flowers both in treated and untreated plants supports the idea that *C. sativa* has intrinsic sexual plasticity that can be accentuated or redirected by the chemical inhibition of ethylene signaling.

### 4.7. Functional Validation: Induced Pollen Is Capable of Fertilization and Seed Production

Despite the heterogeneous anatomical response, STS-induced masculinization produced a subset of flowers capable of completing the male reproductive function. These flowers produced viable pollen, released it through longitudinal anther dehiscence, and fertilized untreated female flowers under open-pollination conditions. The pollen viability of *C. sativa* depends on the plant variety or cultivar, as well as environmental conditions, showing a wide range of values. Wizenberg et al. [62] conducted a study comparing pollen viability among genetically male *Cannabis* plants (unisexual males) and two lines of feminized female plants: one induced by drought (drought-induced cosexual plants) and the other by chemical means (chemically induced cosexual plants). The authors found that unisexual males (36.6–42.3% viability) and drought-induced cosexual plants (37.3–39.9% viability) produced pollen that was 200% more viable than that from chemically-induced cosexual plants (12.4–38.7% viability). In other chemically-induced male plants, the pollen viability observed ranged from 51 to 96% [37,62]. In the present study, pollen viability was 54%, which falls within the range reported for chemically-induced male flowers in *Cannabis* and slightly above the range reported by Wizenberg et al. [63] for unisexual males of the CFX-2 cultivar.

Lubell and Brand [13] suggested that pollen produced by male flowers on genetically female plants can be used to produce exclusively female seeds, even though pollen production may be reduced compared with that of genetically male plants. In our work, the development of mature fruits and fully formed seeds confirmed that at least part of the pollen produced by STS-induced flowers was functional throughout the reproductive process.

This functional validation is essential because pollen staining alone provides only partial evidence of reproductive competence. A pollen grain may stain as viable but still fail to be released, reach the stigma, germinate, grow through the pistil, or complete fertilization. By combining anatomical observations, pollen viability assessment, anther dehiscence, pollen release, and seed production after open pollination with untreated female plants, the present study demonstrates that STS-induced flowers can complete the functional pathway from floral sex reversal to fertilization.

These results agree with previous studies showing that chemically-induced male flowers in genetically female *Cannabis* can produce pollen capable of fertilization and seed production. Ram and Sett [30] demonstrated that STS-induced male flowers produced pollen grains that germinated on stigmas and affected seed sets. Lubell and Brand [13] and Flajšman et al. [24] further showed the practical value of STS for feminized seed production, while Timoteo Junior and Oswald [38] identified STS as one of the most effective ethylene inhibitors for sex reversal in high-THC *Cannabis* cultivars. However, although the seeds obtained in this study showed normal morphology, germination assays would be necessary to confirm that they are viable and capable of producing offspring.

From an applied perspective, these results reinforce the usefulness of STS for feminized seed production in *C. sativa*. However, they also show that STS efficiency should not be assessed solely by the number of externally visible male flowers. A complete evaluation should include inflorescence architecture, floral sexual identity, anther anatomy, progression of microsporogenesis, tapetal behavior, pollen viability, anther dehiscence, pollen release, and seed set.

## 5. Conclusions

STS-induced masculinization in genetically female plants of *Cannabis sativa* cv. ‘Pasionaria S’ occurs through the early redirection of floral meristem identity within a conserved female-patterned inflorescence architecture. Rather than transforming already differentiated pistillate flowers, STS redirects newly formed meristems toward a staminate developmental pathway.

Induced staminate flowers can complete the typical sequence of anther and pollen development and produce functional pollen. However, this response is heterogeneous, including flowers with normal anther development and viable pollen, flowers with disrupted microsporogenesis or collapsed anthers, and occasional mixed flowers with localized androecial expression. Thus, floral sex identity, anther morphogenesis, pollen viability, dehiscence, and fertilization capacity should be considered related but partially independent components of the masculinization response.

The formation of mature seeds after open pollination confirmed the reproductive functionality of at least part of the STS-induced pollen. From an applied perspective, these results provide anatomical and developmental criteria for evaluating and optimizing STS-based masculinization protocols in *Cannabis*. Specifically, the detailed characterization of anther structure, microsporogenesis, pollen maturation, and dehiscence allows distinguishing between flowers that are only externally masculinized and those that are functionally capable of producing viable pollen. This distinction is particularly relevant for quality control in feminized seed production, where the presence of male-looking flowers does not necessarily guarantee reproductive functionality. In addition, these criteria can be used to compare different STS treatments, including dose, timing, and frequency, and to identify conditions that promote complete anther development rather than partial or defective masculinization. These anatomical markers may also support the selection of genotypes with a more consistent response to STS and enable the early diagnosis of developmental failures that are not evident from external male flower formation alone. Overall, STS-induced masculinization in *C. sativa* is best understood as a staged and variable developmental redirection, not as a simple conversion of female flowers into fully functional male flowers.

## Data Availability

The original contributions presented in this study are included in the article.
